# Multi-organ failure caused by lasagnas: A case report of *Bacillus cereus* food poisoning

**DOI:** 10.3389/fped.2022.978250

**Published:** 2022-09-14

**Authors:** Marin Thery, Vladimir L. Cousin, Pierre Tissieres, Maxime Enault, Luc Morin

**Affiliations:** ^1^Pediatric and Neonatal Intensive Care Unit, DMU 3 Santé de L'enfant et de l'Adolescent, Assistance Publique-Hôpitaux de Paris Paris Saclay, Bicêtre Hospital, Le Kremlin-Bicêtre, France; ^2^Pediatric Intensive Care Unit, Département de la Femme, de l'Enfant et de l'Adolescent, Hôpitaux Universitaires de Genève, Genève, Switzerland; ^3^Institute of Integrative Biology of the Cell, CNRS, CEA, Paris Saclay University, Gif-sur-Yvette, France; ^4^Assistance Publique-Hôpitaux de Paris, Pediatric Emergency Department, Armand Trousseau University Hospital, Sorbonne Université, Paris, France

**Keywords:** sepsis, food poisoning, liver failure acute, *Bacillus cereus*, pediatrics, continuous venovenous hemofiltration

## Abstract

We report a *Bacillus cereus*, cereulide producing strain, food poisoning of two sisters. After eating a few bites of pasta cooked 3 days earlier, a 13-year-old girl developed mild symptoms. However, her 11-year-old sister suffered, 40 h after ingestion of the entire platter, a multi-organ failure including acute liver failure, rhabdomyolysis, disseminated intravascular coagulation, and acute kidney injury (AKI). She received supportive care in pediatric intensive care using mechanical ventilation, hemofiltration, and high-doses vasopressors. She was specifically treated for toxin-mediated disease using blood purification and further digestive decontamination. This report highlights the potential severity of *B. cereus* food poisoning but also a successful dual treatment including toxin removal and antimicrobial treatment to prevent toxin production.

## Introduction

*Bacillus cereus* is an aerobic Gram-positive spore-forming bacteria (1). *Bacillus cereus* is described as a foodborne pathogenic bacterium that causes food poisoning of two types: diarrheal and/or emetic syndromes, that both are linked to toxins (2). The diarrheal syndrome is caused by the production of heat-labile proteinaceous enterotoxin complexes (mainly hemolysin BL, NHE, and cytotoxin K) in the small intestine by *B. cereus* cells. Symptoms are abdominal pain with watery diarrhea and occasionally nausea and emesis. The second syndrome, emetic food-borne poisoning, is characterized by rapid onset of symptoms (nausea, vomiting, and abdominal cramping) indicating the presence of preformed toxin in the ingested food (3, 4). This emetic toxin, cereulide, is characterized by its resistance to heat and to most usual hygienic procedures in food processing. The emetic toxin directly acts on the afferent vagus nerve and vomiting center in the central nervous system (3).

Both clinical forms are usually self-limited and recover within 24 h (2).

In all types of *B. cereus* disease, the virulence depends on the strain and is closely linked with toxin production in a dose-dependent manner (1, 5). Both types of food poisoning related to *B. cereus* are generally benign with mild symptoms. However, some more invasive, and lethal cases of rhabdomyolysis and/or liver failure due to the emetic toxin have been reported (6–8). In such cases, mortality is high (50%) and one lifesaving liver transplantation has been described (9).

Here, we report a family food-borne poisoning by a *B. cereus* emetic toxin which lead to two clinical presentations: a multi-organ failure for one family member due to the combination of a toxin-mediated disease and intestinal infection and a mild form for her sister with a food-borne intoxication with the pre-formed toxin. The intoxication was identified early in the course of the disease; hence the patient was not enlisted for liver transplantation, and successfully transitioned to full recovery with hemodynamic support including angiotensin 2 infusion, extra-corporeal blood purification, and further digestive decontamination with enteral vancomycin.

## Case

A previously well 11-year-old girl was admitted to the pediatric emergency department for abdominal pain and emesis. She was with her family in a rental during an especially warm summer (40°C during the day). Symptoms started 20 min after she and her 13-year-old sister ate leftover lasagnas. Both sisters reported the meal was unusually smelly and had a bad taste of “old cheese,” hence the elder stopped eating but not our patient. Both sisters presented abdominal pain and emesis and were brought to the hospital 6 h after the onset of symptoms. Parental interrogation reported the lasagnas had been prepared 72 h earlier and stored in a faulty refrigerator. They presented signs of clinical dehydration, had unremarkable laboratory results, and received intra-venous fluids. The 13-year-old girl's symptoms quickly improved, and she was discharged after 48 h of observation.

Twenty-four hours after admission, our 11-year-old patient complained of chest pain and intense abdominal pain. On clinical examination, she was tachycardic and hypotensive and presented hematemesis. Her laboratory tests revealed a multi-organ involvement with acute kidney injury (AKI), acute liver failure, pancreatitis, rhabdomyolysis, and elevated cardiac enzymes (Table 1). She was referred to our pediatric intensive care unit (PICU) for hepatic transplantation.

**Table 1:** Summary of the evolution of laboratory tests of the patient.

**Date**	**13/08**	**15/08**	**15/08**	**16/08**	**17/08**	**18/08**	**20/08**	**24/08**	**26/08**	**01/09**
**Delay after ingestion**	**H2**	**H30**	**H48**	**H72**	**D4**	**D5**	**D7**	**D11**	**D13**	**D19**
Arterial pH	–	7.26	7.41	7.24	7.30	7.47	7.43	7.37	7.43	–
pCO_2_ (mmHg)	–	35	26	30.6	30.6	28	27	40	34	–
Lactate (mmol/l)	**–**	**12.2**	5.5	8.1	3.0	3.1	1.7	1.0	1.3	–
Hemoglobin (g/dl)	16.3	14.7	12.4	10.8	8.3	9.1	9.7	8.6	7.9	8.5
Platelets (per mm^3^)	385,000	312,000	166,000	107,000	31,000	**16,000**	**16,000**	50,000	88,000	358,000
Sodium (mmol/l)	141	139	137	147	148	141	134	136	135	144
Potassium (mmol/l)	4.3	4.5	**6.9**	4.4	4.0	3.5	4.7	4.3	4.2	3,5
Phosphor (mmol/l)	–	–	0.81	1.69	1.42	2.2	1.39	1.41	1.66	1,44
Creatinine (μmol/l)	45	199	**218**	82	51	44	42	101	170	124
Urea (mmol/l)	–	17	15.4	4.7	3.3	–	1.7	5.9	9.4	14,9
ASAT (U/l)	**–**	**5,912**	1,838	559	410	1,155	1,267	375	229	234
ALAT (U/l)	**–**	**8,571**	5,141	2,736	1,441	1,044	1,138	519	324	237
GGT (U/l)	–	45	41	35	145	91	177	337	708	326
Bilirubinemia T/C (μmol/l)	–	47/28	52/33	25/23	20/17	30/26	42/36	26/21	22/20	12/–
Fibrinogen (g/l)	–	1.5	1.1	1.1	0.9	1.2	1.5	2.5	2.7	3,4
Prothrombin Time (%)	**–**	**19**	22	22	33	–	71	91	>100	>100
Factor V (%)	**–**	**26**	31	37	62	28	100	>100	>100	>100
Creatine kinase (U/l)	–	2,134	2,209	–	–	**36,666**	18,037	1,823	4,568	3,473
Ammonia (μmol/l)	–	269	230	**335**	116	36	38	–	–	–
Stool culture—flora						Absent		Absent	Poor	Normal and rich
Stool culture—presence of B cereus						Many colonies		Many colonies	Some colonies	Absent

Bold values are the most abnormal values of the patient.

**Figure 1:**
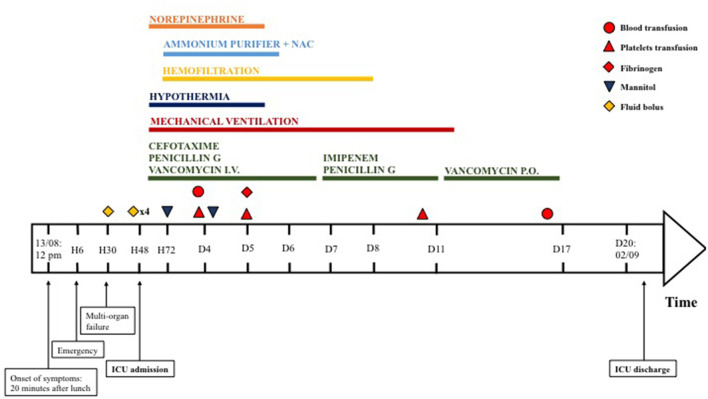
Timeline of treatment for multi-organ failure.

On her arrival in PICU, she was drowsy [Glasgow coma scale (GCS) 13/15—Eye 3/4 Verbal 4/5 Motor 6/6], without respiratory nor hemodynamic failure. The rest of her physical examination was unremarkable, and her blood sugar levels were normal. Six hours after her admission, she suddenly became non-responsive (GCS 8/15) with clinical and trans-cranial Doppler pattern of cerebral edema and hepatic encephalopathy. She required oro-tracheal intubation, mechanical ventilation, osmotherapy, hypothermia, and aggressive hemodynamic support. Laboratory results confirmed hyperammonemia (230 mmol/L), rhabdomyolysis with anuric AKI (creatinine 218 μmol/L), and hyperkalemia (6.9 mmol/L). She was started on sodium benzoate and sodium phenylbutyrate, n-acetylcysteine, and high-flow continuous veno-venous hemodiafiltration (Figure 1). She was treated with extra-corporeal blood purification with an enhanced AN69 hemofilter with increased adsorption capacities of small molecules (oXiris, Baxter Acute Therapies). She was hypotensive and tachycardic despite 60 ml/kg of fluids boluses and high doses of norepinephrine (16 mg/h, i.e., 6.6 μg/kg/min). With this refractory vasoplegic shock, she was started on angiotensin 2 with a dose of 15 ng/kg/min and norepinephrine was gradually withdrawn and stopped on day 6 of ICU admission.

She was initially started on cefotaxime and micafungin according to the local acute liver failure guidelines. The acute liver failure etiological assessment including hepatotropic viruses, toxic, auto-immune hepatitis, Wilson's disease, and mitochondrial disorder was negative. On day 2 of ICU admission, the duty consultant suspected sepsis due to food-borne *B. cereus* toxin-mediated disease. The patient was started on IV vancomycin and benzylpenicillin. Emesis, stool, and blood were sent to the lab and confirmed the diagnosis. *Bacillus cereus* was cultured in stool and emesis. Toxins were evidenced in stool (cereulide and NHE toxins) and emesis (NHE toxin). The antibiogram of the bacteria was usual for *B. cereus*, with resistance to penicillin G and amoxicillin, intermediate resistance to piperacillin, and amoxicillin/clavulanate, and was sensitive to imipenem, gentamicin, macrolides, rifampicin, and cotrimoxazole. Despite 5 days of intravenous vancomycin, the patient had persistent *B. cereus* culture in stool samples and slow improvement of her laboratory in particular creatinine kinase and renal function, both partially masked by the previous use of renal replacement therapy. After discussion with the antimicrobial stewardship team, we changed the antibiotics to imipenem-cilastatin. However, as stool cultures showed numerous *B. cereus* colonies (Table 1) already at Day 5 and again at Day 11, and in view of a rebound in the rhabdomyolysis, we decided to add a 7-day treatment with enteral vancomycin for digestive decontamination. After 5 days of enteral vancomycin, stool cultures became negative (Figure 1), and the laboratory values improved.

Multi-organ dysfunction syndrome gradually regressed with complete neurological and hepatic recovery. The patient was on hemofiltration for 8 days, initially for AKI, blood purification, and hyperammonemia and after day 5 for AKI only. At PICU discharge, the renal function was persistently impaired (creatinine 124 μmol/L and urea 14.9 mmol/L) but did not require further hemodialysis or antihypertensive treatment. The patient was discharged to the ward on day 19 and home on day 40. Her neurological, renal, and hepatic status were normal upon discharge home and 24 months later.

## Discussion

*Bacillus cereus* usually causes mild food poisoning with mild symptoms (10). Enterotoxin food poisoning can present as acute and severe forms, with both emetic and enterotoxin co-present, such as in this case (9). We reported here a case of an 11-year-old patient with multi-organ failure treated with hemodynamic supportive care, blood purification, and digestive decontamination.

*Bacillus cereus* produced a large number of different toxins, both emetic (cereulide) and enterotoxin (hemolysin BL, non-hemolytic enterotoxin, and cytotoxin K). The enterotoxins are produced in the digestive tract by ingested *B. cereus*. These toxins have a different mode of action through pore formation and other forms of lesion of the enterocytes barriers (3). The emetic toxin, cereulide, is a dodecadepsi-peptide highly resistant to heat, and acidic conditions, hence it is not altered by gastric acid nor reheating foods stored at ambient temperature (3, 11). When pre-formed in the food and ingested, cereulide reaches the stomach and duodenum and causes emesis by binding the 5-HT3 receptor and causing a stimulation of the vagus afferent nerve. The invasion time of our patient (20 min) is consistent with the literature (12) but in our case, the clinical deterioration occurred significantly later compared to published cases (6, 8, 9, 13). Cereulide also causes alteration of the mitochondrial activity by inhibition of fatty acid oxidation, cellular damage (14), and inhibition of human natural killer cells (15). The specific detection of toxin-producing *B. cereus* strains can be done by several tools such as polymerase chain reaction, mass spectrometry and spectroscopy, or immunoassay of cereulide synthetase (3, 16).

Acute liver failure and rhabdomyolysis are almost constant biological abnormalities that are explained by the alteration of mitochondrial function caused by the toxin (10, 14). Both could lead to AKI. Some patients, including ours, can develop multiple organ failures with liver failure and encephalopathy, DIC, hypoglycemia, AKI, and metabolic acidosis. This pattern could be similar to a toxic Reye's syndrome (17). Hence, the detection of *B. cereus* and cereulide is important to correctly diagnose this pathology before its progression to multi-organ failure and death. In our case, the rapid diagnosis suspicion, and the literature review of the spontaneous recovery of *B. cereus* toxin-mediated liver failure helped the liver transplant team with the decision to post-pone liver transplantation. In the absence of known reversible etiology, similar patients are usually enlisted to national priority for liver transplantation and receive an organ in a 24–48 h window.

The 13-year-old sister who presented a benign form only ingested a few bites of her meal. This likely indicates that the ratio of ingested pre-formed toxin to body weight is proportional to the severity of the induced illness. However, direct measurement of plasma cereulide levels is difficult and not standardized. This has been suggested by Shiota et al. and they proposed to determine the values of cereulide by using a HEp-2 vacuolation assay (13). An unexplained particularity of this disease is the fact that all reported patients were teenagers or young adults.

In the context of a severe, food-borne induced, toxin-mediated disease, we based our treatment on three arms: hemodynamic supportive care including angiotensin 2 infusion, blood purification, and bacterial decontamination. Blood purification therapy is an option that has been described for one patient (13). Cereulide is present in stool, blood, and urines and is presumed to be eliminated in the urine. Thus, hemodiafiltration could help clear the toxin, especially in the case of AKI. Cereulide also binds strongly to plasma proteins; plasma exchanges could therefore logically be an alternative for patients without hemofiltration indication (13). The Cereulide toxin is a 1.2 kDa that is theoretically entirely dialyzed by this hemofilter. This strategy aims to reduce the quantity of circulating toxins and improve organ failures. The patient's liver function initially improved and collapsed again following toxin liberation.

*Bacillus cereus* food poisoning is a toxin-mediated disease, not an invasive bacterial infection (3) and antimicrobial therapy is usually not indicated (18). However, with the severity of the clinical situation and the relapse in the symptoms, we decided to treat our patient with intravenous vancomycin and penicillin G to target both *B. cereus* and its toxins. Later, given the continuous increase in creatine kinase levels, persistence of kidney failure, and the absence of *B. cereus* in the patient's blood, we aimed to directly target the toxin production through the treatment of the inoculum (stool culture which showed a pure culture of *B. cereus*). We hypothesized that the bacteria in the gut were producing toxins and that the reduction in bacterial inoculum would decrease that production. After discussion with our antimicrobial stewardship team, we choose to use oral vancomycin as it is a standard treatment for *C difficile* colitis with good tolerance and safety and to monitor the amount of *B cereus* colonies to adapt the duration of treatment. This treatment was not associated with an increase in the toxin burden through bacterial death and led to the absence of *B. cereus* in the feces and the re-appearance of normal flora. To our knowledge, this approach had never been described before.

In summary, we report a case of severe food poisoning caused by the preformed emetic toxin produced by *B. cereus*, which led to multi-organ dysfunction syndrome. The diagnosis was rapidly confirmed with culture and specific PCR in emesis and stool. This report emphasizes the need for pediatric intensivists to know this pathology since there is a particular treatment and a potentially reversible cause. We also evidenced the association between toxin quantity ingestion and disease severity. Finally, we propose the combination of blood purification and digestive tract decontamination with enteral vancomycin as a new insight for treatment in the most severe cases with combined intestinal contamination and toxin-mediated disease.

## Data availability statement

The original contributions presented in the study are included in the article/supplementary material, further inquiries can be directed to the corresponding author/s.

## Ethics statement

Written informed consent was obtained from the minor(s)' legal guardian/next of kin for the publication of any potentially identifiable images or data included in this article.

## Author contributions

All authors contributed to the care of the patient, the draft and modification, and approve of the final version of the manuscript.

## Funding

Open access funding was provided by the University of Geneva.

## Conflict of interest

The authors declare that the research was conducted in the absence of any commercial or financial relationships that could be construed as a potential conflict of interest.

## Publisher's note

All claims expressed in this article are solely those of the authors and do not necessarily represent those of their affiliated organizations, or those of the publisher, the editors and the reviewers. Any product that may be evaluated in this article, or claim that may be made by its manufacturer, is not guaranteed or endorsed by the publisher.
