# Erratum: Multi-organ failure caused by lasagnas: A case report of *Bacillus cereus* food poisoning

**DOI:** 10.3389/fped.2023.1178208

**Published:** 2023-03-17

**Authors:** Frontiers Production Office

**Affiliations:** Frontiers Media SA, Lausanne, Switzerland

**Keywords:** sepsis, food poisoning, liver failure acute, *Bacillus cereus*, pediatrics, continuous venovenous hemofiltration

An Erratum on Multi-organ failure caused by lasagnas: A case report of *Bacillus cereus* food poisoning By Thery M, Cousin VL, Tissieres P, Enault M and Morin L. (2022) Front. Pediatr. 10:978250. doi: 10.3389/fped.2022.978250

An omission to the funding section of the original article was made in error. The following sentence has been added: “Open access funding was provided by the University Of Geneva”.

The original version of this article has been updated.

